# Primary Myiasis in Surgical Wound of Mandible Symphisis Fracture

**DOI:** 10.1155/2019/5393405

**Published:** 2019-12-30

**Authors:** Gustavo A. Lauand, Felipe G. G. P. Lima, Ricardo P. da Silva, Larissa Rodrigues Santiago, Jonas B. Dantas, Cláudia J. Silva, Lair M. Furtado

**Affiliations:** Clinical Hospital of the Federal University of Uberlândia, Uberlândia, Brazil

## Abstract

A 42 year old patient was referred to the Department of Oral and Maxillofacial Surgery of the Federal University of Uberlândia, for treatment of mandibular fractures (condyles and symphysis), a victim of a run over. The symphysis was surgically approached, using as surgical access the pre-existing laceration in the submental region. Five days after discharge, the patient returned with dehiscence of the wound and physical examination showed infestation by larvae in the symphysis. Mechanical removal and debridement were performed under local anesthesia, where plate exposure was noted. The patient underwent oral ivermectin therapy, intravenous antibiotic therapy and a thorough debridement was performed under general anesthesia due to the invasion of deep spaces in the supra-hyoid region. After 2 weeks, it presented with purulent drainage at the site. The miniplates were replaced by a 2.4 mm reconstruction plate and antibiotic therapy was maintained. Due to the social risk, the patient remained hospitalized for 45 days, when he was discharged with outpatient return, but did not attend the returns.

## 1. Introduction

Myiasis is a parasitic disease caused by certain fly larvae at some stage of their development, in which human and animal tissues form part of their diet [1] and can be classified as primary and secondary. In the primary, the larvae feed on living tissue, and on the secondary tissue necrotic tissue [2]. Socioeconomic conditions are associated with disease risk factors, including advanced age, lack of hygiene, previous diabetes, housing in rural areas, homelessness, neurological diseases, and low purchasing power [3]. It affects many regions of the body: nasal mucosa, ear canal, urogenital mucosa, and may occur associated with other pathologies such as squamous cell carcinoma in the oral region, or in traumatized areas [1, 2, 4–6]. Its treatment is generally performed by debridement, larvae and devitalized tissue removal associated with the administration of ivermectin and antibiotics [7, 8]. The aim of this study was to present a case of myiasis in symphisis wound post-reduction and fixation of mandibular fracture.

## 2. Case Report

A patient, 42 years old, was referred to the Clinical Hospital of the Federal University of Uberlândia, with face trauma due to run over. In the anamnesis, the patient declared himself a smoker, a chronic alcoholic, and had a psychiatric condition. He claimed to live in shared residence with other individuals also dependent on drugs and in precarious sanitary conditions. The preoperative laboratory tests showed no alterations.

**Figure 1:**
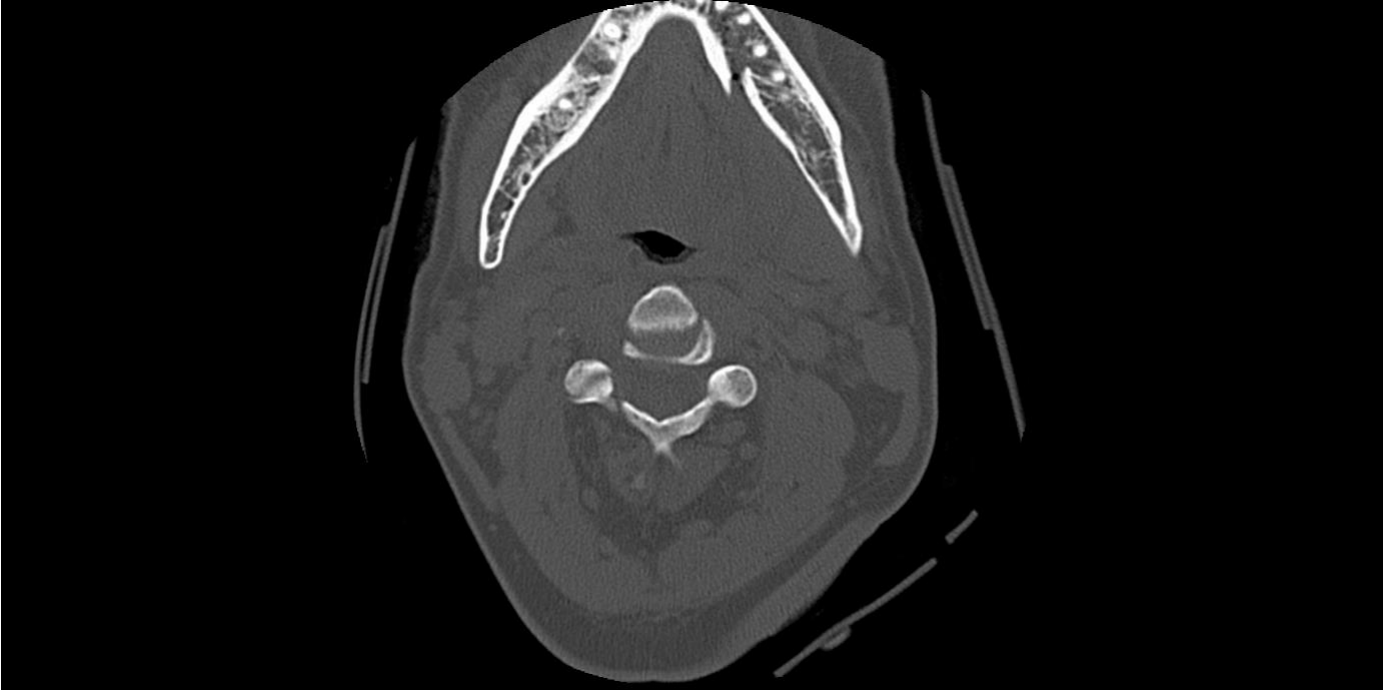
Axial section of computed tomography revealing fracture of mandibular symphysis.

**Figure 2:**
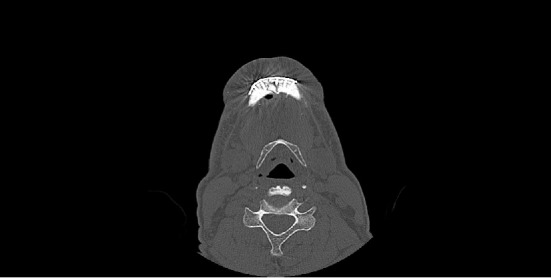
Rigid internal fixation of mandibular symphysis fracture with 2.0 system miniplates.

During the clinical examination, signs suggestive of mandibular fractures were observed, which were confirmed by imaging examinations, closing the diagnosis of symphysis and mandibular condyles fracture (Figure 1). The symphysis was surgically treated, since condylar fractures were high and contraindicated for miniplate fixation. Pre-existing laceration in the submental region was used as surgical access. Surgery was performed without complications and the patient was discharged after 24 hours, referred home with oral antibiotic therapy (amoxicillin 500 mg, 8/8 hours for 7 days) and scheduled outpatient return (Figure 2).

**Figure 3:**
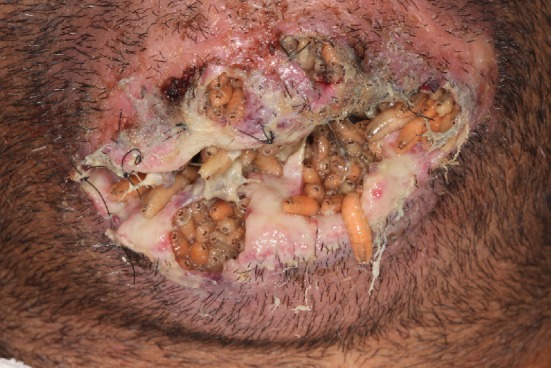
Clinical view from the primary surgery, showing larval infestation.

After five days, he returned with dehiscence in the surgical wound and the clinical examination revealed the presence of larvae in the symphisis (Figure 3). Patient presented with poor personal hygiene and reported not having performed the dressing changes in the surgical wound, remaining with the same dressing for the 5 consecutive days. Also, did not carry out the use of prescribed medications.

**Figure 4:**
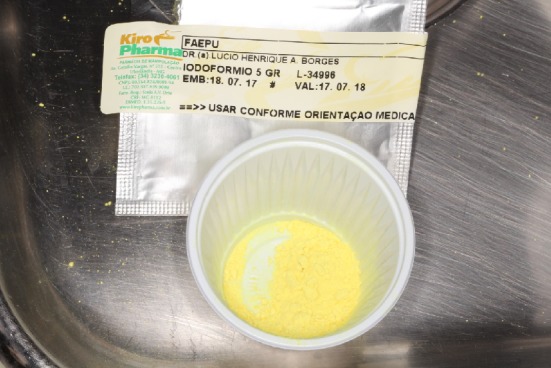
Iodorform compound used topically to facilitate the larval removal.

**Figure 5:**
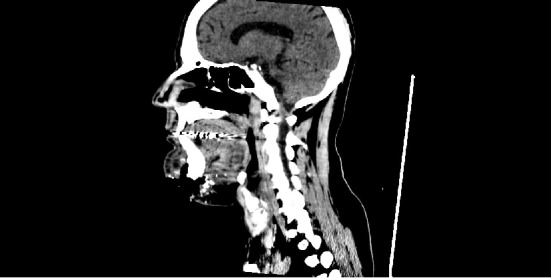
Sagital section of computed tomography contrast enhanced, revealing extensive damage to the suprahyoid soft tissues.

It was proposed to remove the larvae under local anesthesia and surgical exploration of the area. During the procedure, approximately 250 larvae were removed and topical iodoform was applied (Figure 4). The exposure of the symphysis fracture fixation plates was observed, in addition there still was the presence of larvae in deeper tissues that could not be removed in this first stage with local anesthesia. A computed tomography was performed for suprahyoid space examination, due to the extensive tissue destruction and the impossibility of evaluation of deeper larvae (Figure 5). Ivermectin was administered orally (6 mg/day, for 3 days), intravenous antibiotic therapy (IV) with ampicillin + sulbactam 2 g, 6/6 hours for 7 days, and the larval remnant was removed under general anesthesia.

**Figure 6:**
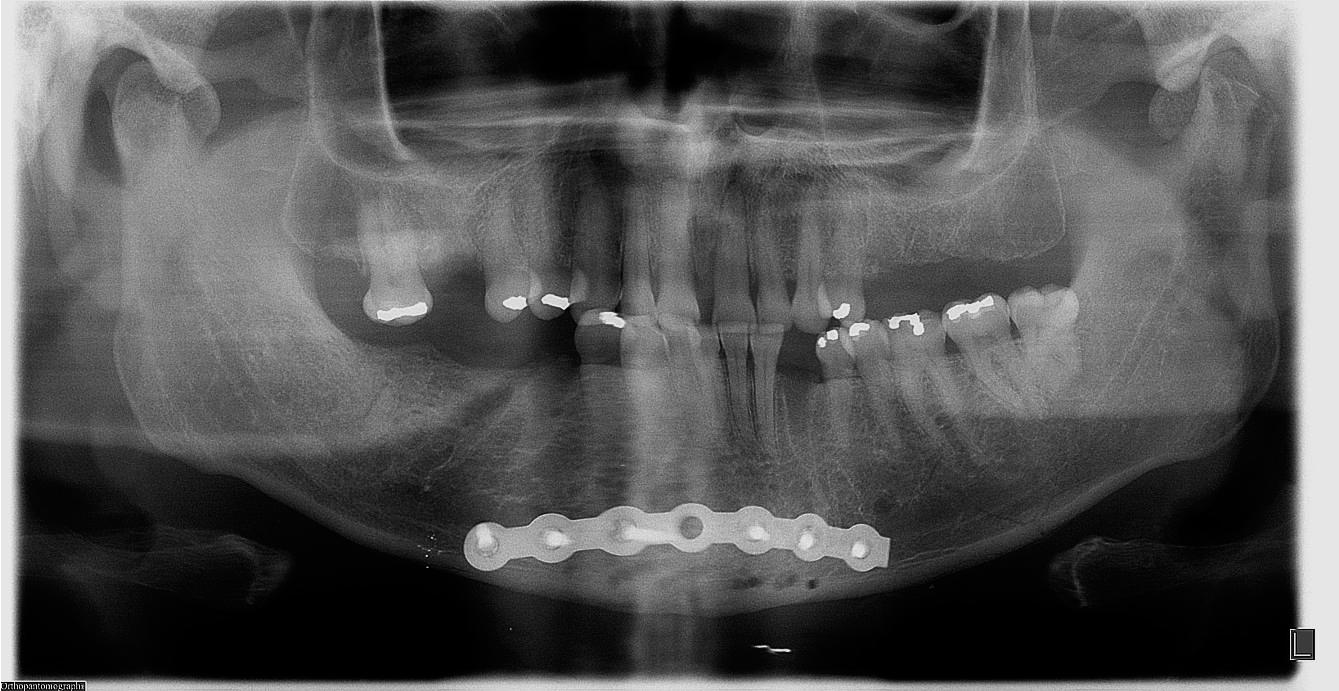
Ortopantomogram radiography showing internal rigid fixation with 2.4 mm reconstruction plate.

**Figure 7:**
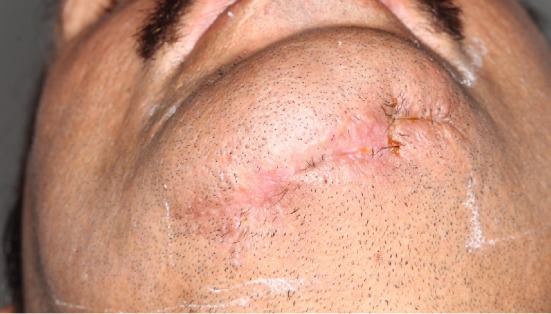
Final clinical aspect, with complete wound healing.

After 2 weeks, the wound evolved with purulent drainage. The miniplates were replaced by a 2.4 mm reconstruction plate and again the patient underwent antibiotic therapy (IV) (Figure 6). The patient remained hospitalized for nursing care and was discharged 45 days after admission to the psychiatric ward and did not attend the return. Despite this, there was remission of the condition and complete closure of the wound in 15 days, after the last surgical intervention, without significant sequelae (Figure 7).

## 3. Discussion

Myiasis is an aggressive condition, more common in some specific patients and endemic in tropical countries. Its diagnosis is based on visual clinical findings of larvae deposited in tissues, either on the surface of the skin or in cavities (nose, mouth, orbit, ear, anus, vagina and penis); or based on images and symptoms, when on internal organs [4, 8–10].

Some authors demonstrate the correlation of the disease with several risk factors. Durão et al. (2016) reported digital myiasis in a patient with psychiatric disorder [11]; Giri et al. (2015) reported cerebral myiasis in a patient which neglected follow-up consultations [12]; Manjunath and Pinto (2018) reported nasal myiasis in a diabetic patient who presented with recurrent myiasis [13]; Antunes et al. (2011) reported a case series of 6 patients that presented psychologic or neurological disorder [14]. At the initial clinical examination, we observed extensive dehiscence in the surgical wound, tissue destruction, necrosis, and a large amount of larvae. There was no compliance to the follow-up consultations and neither wound care nor cleanse, predisposing the disease development. The alcoholic behavior and drug addiction in this case associated with low socioeconomic status played a major role in the disease curse.

The first approach consisted in removing the larvae, mostly mechanically. The area was debrided and computed tomography performed to investigate involvement of deep cervical spaces. The antibiotic regimen based on penicillin was established in this case, aiming to prevent secondary infections due to lesion extension and necrosis. Although there is no consensus in theliterature about it, some authors reported myiasis resistance to the treatment of 2 grams of oxacillin over the 7 day period [15]. Ceftriaxone at a dose of 1 g twice a day associated with the treatment of debridement and removal of necrotic tissue proved to be efficient in curing the patient [8]. The existence of various antibiotic therapies increases the surgeon's options, so their choice varies according to their experience and drug availability.

For treatment of larvae, ivermectin 6 mg once daily for 3 days as antiparasitic therapy was chosen. Ivermectin belongs to the macrolide family and acts by blocking impulses in the nerve endings by the action of gamma-amino-butyric acid, paralyzing the larvae. Acetylcholine, a mammalian neurotransmitter, is not affected by this drug, ensuring its efficacy. The dosage varies according to the literature. Hassona et al. (2014) [16] indicates the use of a single dose of 150–200 micrograms/kg body weight. Shinohara et al. (2004) [17] reported using 6 mg in 2 doses at 24-hour intervals between doses.

As a drug protocol, 18 mg was used, approaching 15 mg for a 75 kg person, as indicated by Hassona et al. (2014). Ampicillin 2 g associated with Sulbactam EV, every 6 hours for 7 days, was used for the prophylaxis of secondary infections, due to larval remnants. Topical therapy was also used with iodoform diluted in 0.9% physiological solution, in an attempt to create asphyxia of the surface of the larvae, aiding its removal. Its use, however, is contradictory in the literature and not standardized, with several substances available for this principle, such as oil of turpentine, ether and alcohol [17].

Patient evolved with infection 15 days after the first hospitalization, requiring reintervention under general anesthesia for the replacement of the miniplates of fixation by a reconstruction plate 2.4 mm. He was again submitted to empirical therapy with Ampicillin 2 g associated with Sulbactam for another week, with complete healing of the wound. Dressings were maintained with topical gentamicin (nebacetin) in ointment and daily cleansing with 2% chlorhexidine. There was total remission and complete healing.

The smear and culture results were negative for Baar culture and were available after the healing period, thus revealing that the empiric antibiotics were efficient. The larvae were not identified, but epidemiologically, the larvae that mostly affect the southeast region of Brazil (Minas Gerais-MG) are usually classified as *Cochliomyia hominivorax* (Diptera: Calliphoridae) [18].

The local health care system, although having a social support, it is usually performed during the period of hospitalization of the patient. The cooperation to the outpatient treatment is assigned to the patient or a guardian/companion, making adherence to treatment protocols difficult, and making the compulsory hospitalization of the patient in question at the psychiatric ward.

## 4. Conclusion

Myiasis is a larval condition that requires immediate surgical treatment because of its rapid tissue damage and wide extension. Usually, it affects some predisposed patients. In this case, the psychiatric condition, alcohol behavior and drug addiction were the main factors implied in the nonpatient's non-compliance to the consultants and wound care. Furthermore, it implies in delayed healing, which also predisposes wound contamination and larval deposits. The proposed treatment and empiric antibiotic chosen, were effective in the resolution of the disease.
